# Supplementary material for: Mathematical Modeling of Salmonella Cancer Therapies Demonstrates the Necessity of Both Bacterial Cytotoxicity and Immune Activation
Source: Bioengineering (Basel). 2025 Jul 10;12(7):751. doi: 10.3390/bioengineering12070751 (PMC12293069; doi:10.3390/bioengineering12070751)
Supplement: Supplementary file 1 [file bioengineering-12-00751-s001.zip › bioengineering-3674668-supplementary.pdf]

Supplemental Information

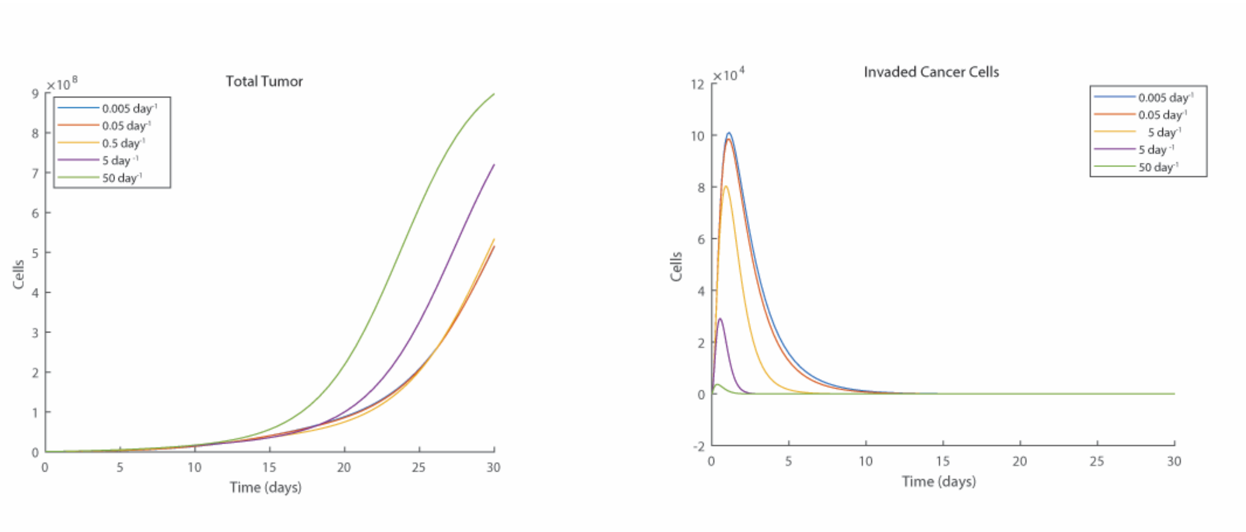

Supplemental Figure 1: The addition of bacterial clearance from the blood, $\frac{dS_B}{dt} = -k_1 S_B - k_{exit} S_B$, without entering the tumor was modeled at rates, k_{exit}, ranging from 0.0005 day^{-1} to 50 day^{-1}. With all other equations remaining constant. The additional method of bacterial clearance did not impact total tumor volume or invaded cancer cells until at a rate beyond physiologically relevant.

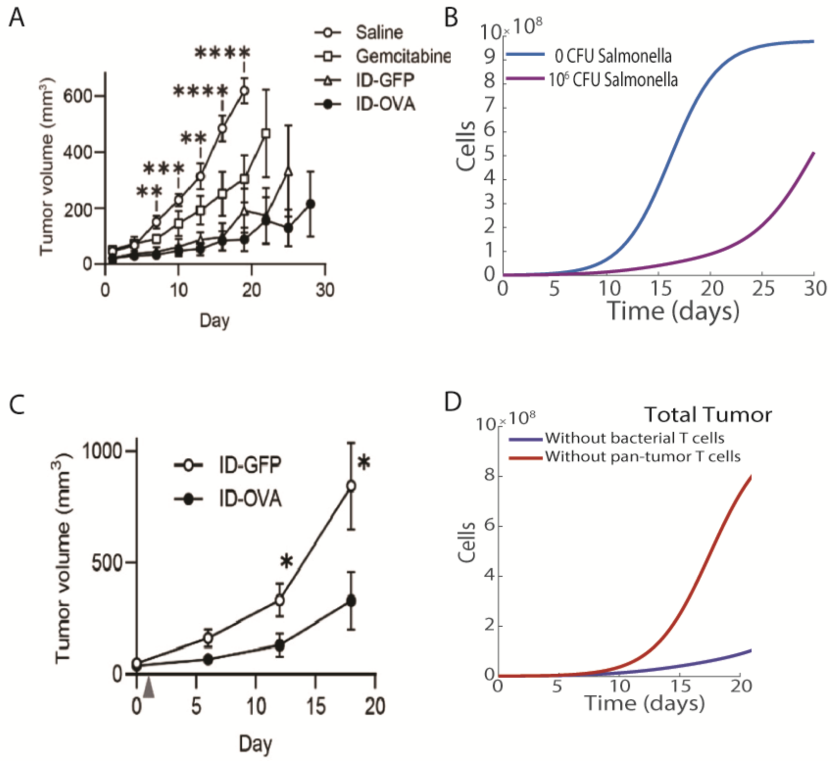

Supplemental Figure 2: Modeling data (B,D) was compared to *in vivo* results (A,C) [1]. A,B) Treating tumors with Salmonella decreased tumor growth in qualitatively similar ways. C,D) The lack of bacterial specific T cells, here represented by ovalbumin delivering Salmonella in an OT-1 mouse reduced tumor growth more than non-specific Salmonella.

1. Raman, V.; Howell, L.M.; Bloom, S.M.K.; Hall, C.L.; Wetherby, V.E.; Minter, L.M.; Kulkarni, A.A.; Forbes, N.S. Intracellular Salmonella Delivery of an Exogenous Immunization Antigen Refocuses CD8 T Cells against Cancer Cells, Eliminates Pancreatic Tumors and Forms Antitumor Immunity. *Front. Immunol.* **2023**, *14*, doi:10.3389/fimmu.2023.1228532.
